# Predicting Cohort-Specific Cervical Cancer Incidence From Population-Based Surveys of Human Papilloma Virus Prevalence: A Worldwide Study

**DOI:** 10.1093/aje/kwab254

**Published:** 2021-10-15

**Authors:** Rosa Schulte-Frohlinde, Damien Georges, Gary M Clifford, Iacopo Baussano

**Keywords:** forecasting, papillomavirus infections, uterine cervical neoplasms

## Abstract

Predictions of cervical cancer burden and the impact of measures taken to control this cancer are usually data-demanding and based on complex assumptions. We propose a predictive method (called PANDORA) based on human papillomavirus (HPV) prevalence, measured 1993–2008, and cervical cancer incidence (CCI), measured 1993–2012, in the same birth cohorts from different worldwide locations, informed by data on age at detection of high-risk HPV and sexual debut. The model can predict CCI among high-risk HPV–positive women and predict CCI up to 14 years following high-risk HPV detection. We found CCI to increase during the 14 years following high-risk HPV detection in unscreened women aged <35 years but to remain mainly constant among women ≥35 years. Age at sexual debut was a significant modifier of CCI. Using our model, we accurately reproduced CCI among high-risk HPV–positive women as observed in cohort studies and in the general population of multiple countries. We also predicted the annual number of cervical cancer cases and CCI in locations with HPV prevalence data but no cancer registry. These findings could inform cervical cancer control programs in settings without cancer registries, as they can be used to predict future cervical cancer burden from population-based surveys of HPV prevalence.

## Abbreviations

CI: confidence interval
CI5: Cancer Incidence in Five Continents
HPV: human papillomavirus
IARC: International Agency for Research on Cancer
PI: prediction interval

The World Health Organization has recently called to eliminate cervical cancer as a public health problem. The main components of the global strategy are human papillomavirus (HPV) vaccination, cervical cancer screening, and the management of detected disease (1). An accurate quantification of cervical cancer burden is essential to inform the planning of cancer control programs and to monitor the impact of measures to control this cancer. The most valid estimates of cancer burden are obtained from population-based cancer registries, which are, however, resource-demanding. On a global scale, about one-third of countries (65 countries) have high-quality national (or subnational) data on cancer incidence (2), but this coverage, although improving in low- and middle-income countries, remains mostly confined to high-income countries. Hence, current and future trends in cervical cancer incidence in most low- and middle-income countries are, and will remain, unknown. Furthermore, to provide worldwide estimates of cancer burden, the International Agency for Research on Cancer (IARC) regularly compiles the GLOBOCAN database, part of the Global Cancer Observatory (https://gco.iarc.fr/), where sex- and age-specific estimates of incidence and mortality for 38 cancer sites in 185 countries or territories are calculated, relying upon the best available data on cancer incidence and mortality nationally (3).

Mathematical (4) and statistical (5) models are extensively used to predict cervical cancer burden and to project the impact of measures to control this cancer in the absence of high-quality local data. However, prediction validity relies on strong biological and behavioral assumptions, often difficult to assess, and high-quality data, often unavailable. For example, average time between HPV infection acquisition and cervical cancer occurrence, essential to predict cervical cancer incidence, remains difficult to assess, first because it is far more feasible to obtain data on prevalence than on incidence of HPV, and also because follow-up of precancerous lesions without treatment, to understand cervical cancer incidence, is unethical (6). This knowledge gap may affect the validity of cervical cancer risk estimates and of the predicted impact of preventive measures in specific populations. To partially overcome this limitation, we have developed a parsimonious method (named PANDORA) to predict cervical cancer incidence from population-based HPV prevalence data.

### METHODS

Our cervical cancer incidence predictions in the general population are based on estimates of cervical cancer incidence rates in high-risk HPV–positive women, obtained by assessing at an ecological level the association between high-risk HPV prevalence and cervical cancer incidence rates in the same birth cohorts. To estimate our predictions, we also accounted for the effect of the time interval between high-risk HPV prevalence measurement and cervical cancer detection (hereafter “time lag”), of average age at sexual debut within the birth cohort, and of cervical cancer screening. Below we describe data sources and the statistical methods used to parameterize and validate the PANDORA predictive model.

### Data sources

Age-stratified and population-based high-risk HPV prevalence data were obtained from the IARC HPV Prevalence Surveys database (Web Figure 1, available at https://doi.org/10.1093/aje/kwab254). All cross-sectional surveys (*n* = 28) used a standardized protocol for population-based recruitment and detection of HPV in cervical cell samples using a general primer (GP) 5+/6+-based polymerase chain reaction assay detecting at least 36 types (7). The following HPV types were defined as high-risk types: 16, 18, 31, 33, 35, 39, 45, 51, 52, 56, 58, 59, and 68.

Cervical cancer incidence data for the same birth cohorts enrolled in the HPV prevalence surveys were extracted from cancer registries included in Cancer Incidence in Five Continents (CI5), Volumes VIII–XI, a series of monographs published every 5 years by IARC and the International Association of Cancer Registries, including age-stratified data from high-quality cancer registries worldwide (2, 8–10).

### Association between high-risk HPV prevalence and cervical cancer incidence

As a preliminary analysis, we quantified the association between high-risk HPV prevalence and cervical cancer incidence in the same birth cohorts, as reported in CI5, Volume XI (2008–2012) (2), to assess the consistency of the above-mentioned data with the observation that prevalent HPV infections in older women are more likely to be persistent and have a higher risk of causing cervical cancer (11). High-risk HPV prevalence and cervical cancer incidence data in the same birth cohorts were available for 17 locations (Web Figure 1, Web Table 1) and were used to describe the age-specific effect of high-risk HPV prevalence on cervical cancer incidence.

For each location, we calculated the time lag between high-risk HPV prevalence measurement and cervical cancer detection, stratifying the populations into 10-year age groups (age at HPV detection, in years: 25–34, 35–44, 45–54, and 55–64). Subsequently, we assessed the association between age-specific high-risk HPV prevalence and cervical cancer incidence for the same 10-year birth cohort fitting a Poisson regression model, with a linear link function, separately for locations with a time lag <10 and ≥10 years between assessment of high-risk HPV prevalence and cancer incidence. Since HPV prevalence cannot be negative, the intercept of the regression models, which can be interpreted as the cumulated risk of cervical cancer in the time elapsed between the assessment of high-risk HPV prevalence and cancer incidence, was not allowed to be negative. In the Poisson models, we also included age at HPV detection as a modifier of the effect of HPV prevalence on cervical cancer incidence rates. Since cervical cancer screening is an important confounding factor at a population level, we also conducted a sensitivity analysis excluding data from the locations with organized screening programs with reported coverage above 50% (i.e., Amsterdam, the Netherlands, and Turin, Italy, hereafter “locations with screening” (12)). The other locations were considered to be “locations without screening” for the purpose of the present analysis (13, 14). We measured the pseudo-*R*^2^ to assess the goodness of fit of the models (15). See Web Appendix 1 for more details.

### **Cervical cancer incidence among high-risk HPV**–**positive women**

We went on to estimate cervical cancer incidence among high-risk HPV–positive women. In this analysis, we included a subset of 16 locations (Web Table 1) for which cervical cancer incidence data from CI5 Volumes VIII–XI (2, 8–10) covered relevant time intervals, to calculate country-standardized and cohort-specific cervical cancer incidence rates in high-risk HPV–positive women (only Ho Chi Minh City, Vietnam, was excluded). We stratified our populations into the age groups of 20–24, 25–34, 35–44, and 45–54 years at HPV detection. For each location and year since HPV measurement, we calculated the cohort-specific number of high-risk HPV–positive women and cervical cancer cases and computed the cohort-specific incidence of cervical cancer in high-risk HPV–positive women (Web Figure 1).

To estimate cohort-specific incidence of cervical cancer in women with prevalent high-risk HPV infection, we fitted, for each age-group at HPV detection, a mixed-effect linear regression model with years since HPV detection, average age at sexual debut, their interaction, and screening implementation status as covariates, location as grouping variable, and the risk of cervical cancer (on a log scale) as an outcome. See Web Appendix 1 for more details.

To be able to include at least 3 locations in each analysis, we limited the time interval for cancer incidence estimates to 14 years. For the same 14 locations without screening, average age at sexual debut (Web Table 2) was estimated from the IARC HPV Prevalence Surveys database (7), whereas for Amsterdam and Turin it was obtained from national population-based surveys (16, 17). To minimize the impact on our findings of HPV prevalence estimates based on few HPV infections, we also performed a sensitivity analysis restricted to 14 locations in which HPV prevalence was estimated on at least 10 infections in each age group (i.e., we excluded Barcelona, Spain, and Songkla, Thailand).

To assess the proportion of variance explained by location as a grouping variable, we estimated the intraclass correlation coefficient of each model. To assess the internal validity of our model, we estimated the sensitivity of our estimates to data perturbation by computing *R*^2^ using a random 10-fold cross-validation procedure (18). We also assessed the ability of our PANDORA model to predict cervical cancer incidence in a new location based on age-specific high-risk HPV prevalence, average age at sexual debut, and screening by estimating *R*^2^ using a blocked cross-validation procedure (18). To assess the external validity of our approach, we estimated cervical cancer incidence (along with corresponding 90% prediction intervals (PIs)) after 14 years of follow-up among 30- to 59-year-old HPV-positive women and compared our model-based findings with results from 2 cohort studies, with a follow-up of 14 years, designed to quantify the risk of cervical cancer in cohorts of HPV-positive and cytology-negative women (19, 20). Furthermore, for some countries (i.e., Bhutan, Nigeria, and Georgia), we could validate our estimates with data from matched birth cohorts reported by the national population-based cancer registries but which are not included in CI5 (21–23). Last, for the remaining countries without high-quality cancer incidence data, we compared PANDORA estimates of cervical cancer incidence 5 years after the implementation of the surveys, with incidence estimates from GLOBOCAN 2020, which were based on local cancer registry data but not necessarily population-based nor contemporary.

All analyses were performed using R (version 3.6.1; R Foundation for Statistical Computing, Vienna, Austria).

## RESULTS

**Figure 1:**
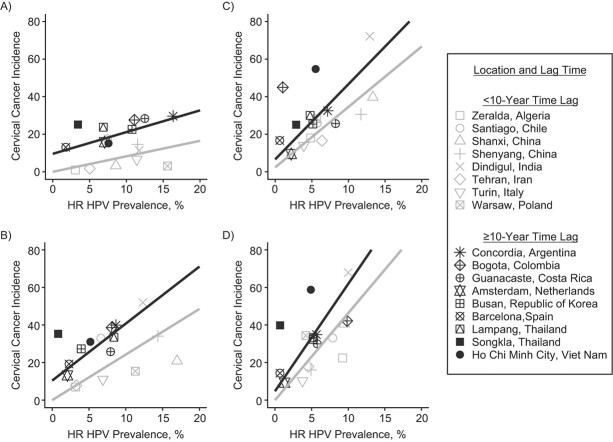
Birth cohort–specific high-risk human papillomavirus (HR HPV) prevalence and cervical cancer incidence rates, multiple countries. Results are shown by age group at HPV detection, time lag between HR HPV prevalence measurement (1993–2008; International Agency for Research on Cancer HPV Prevalence Surveys) and cervical cancer detection (2008–2012; Cancer Incidence in Five Continents Volume XI), and location. The lines represent effect estimates obtained fitting a Poisson regression model. Cervical cancer incidence rate is given per 100,000 woman-years. A) Ages 25–34 years; B) ages 35–44 years; C) ages 45–54 years; D) ages 55–64 years.

Figure 1A–D shows, for 17 locations, cervical cancer incidence rates per 100,000 women plotted against high-risk HPV prevalence, and the estimated increase in cervical cancer incidence per 100,000 women-years per 1% increase in high-risk HPV prevalence, by age group at HPV detection and time lag between assessment of high-risk HPV prevalence and cancer incidence. Estimated cervical cancer incidence increased with high-risk HPV prevalence in all age groups. The size of the effect of HPV prevalence on cancer incidence rates increased with age and was also systematically higher in locations with a longer time lag between HPV detection and cervical cancer assessment in the same birth cohorts (Table 1). The model’s intercept in locations with a time lag ≥10 years decreased from 9.6 (95% confidence interval (CI): 7.2, 11.9) for women aged 25–34 years to 4.8 (95% CI: 3.2, 6.3) for women aged 55–64 years. In locations with a time lag <10 years, the intercept did not significantly differ from zero in any age group, except for showing an intercept of 2.4 (95% CI: 0, 5.9) for women aged 45–54 years. On average, the pseudo-*R*^2^ was 0.61, ranging between 0.49 and 0.81 (Table 1). In a sensitivity analysis excluding locations with screening, estimates were not materially different for locations with time lag of <10 years, whereas, for locations with time lag ≥10 years, the model’s intercept increased with age, and the size of the effect of HPV prevalence on cancer incidence rates was less pronounced (Web Table 3).

**Table 1:** Estimated Increase in Cervical Cancer Incidence per 100,000 Women-Years per 1% Increase in High-Risk Human Papillomavirus Prevalence by Age at Human Papillomavirus Detection and Time Lag Between Human Papillomavirus Prevalence (1993–2008; International Agency for Research on Cancer HPV Prevalence Surveys) and Cancer Incidence (2008–2012; Cancer Incidence in Five Continents Volume XI) Measurement, Multiple Countries

**Age Group, years, and Time Lag, years**	**Intercept**	**95% CI**	**Estimated Increase**	**95% CI**	**Pseudo-*R*** ^**2**^
Ages 25–34					
<10	0	0, 1.5	0.8	0.7, 1.0	0.81
≥10	9.6	7.2, 11.9	1.2	0.9, 1.4	0.49
Ages 35–44					
<10	0	0, 2.4	2.4	2.2, 2.7	0.66
≥10	10.4	8.9, 11.9	3.0	2.6, 3.4	0.56
Ages 45–54					
<10	2.4	0, 5.9	3.2	2.8, 3.7	0.74
≥10	6.6	4.9, 8.4	4.0	3.5, 4.5	0.34
Ages 55–64					
<10	0	0, 5.1	4.6	3.6, 5.6	0.62
≥10	4.8	3.2, 6.3	5.7	5.1, 6.3	0.65

Abbreviation: CI, confidence interval.

Results of the mixed-effect regression model are shown in Table 2. We report, for each age group at time of HPV testing and for average age at sexual debut set at ages 17, 20, and 23 years, the cervical cancer incidence rate per 1,000 HPV-positive women at time of HPV testing (the intercept of the model) and the increased percent in cervical cancer incidence rate in HPV-positive women for every additional year since HPV testing (the slope of the model). Time lag between high-risk HPV prevalence and cancer incidence assessment significantly affected cervical cancer risk in women below 35 years of age and was also significantly modified by the average age at sexual debut. Above age 35, however, cervical cancer risk did not increase significantly with time lag but displayed a negative time trend above age group >45 for age at sexual debut <23 years of age. The cervical cancer incidence rate per 1,000 HPV-positive women increased significantly from the youngest to the oldest age group regardless of age at sexual debut. Cervical cancer screening approximately halved cervical cancer incidence in any age group, although not significantly. The intraclass correlation coefficient (i.e., the proportion of variance explained by setting location as grouping variable) increased with age from 0.67 in 20- to 24-year-olds to more than 0.95 in 45- to 54-year-olds. The average *R*^2^ values for cross-validation were 0.90 and 0.67 using the random and blocked procedures, respectively (Web Table 4). Similar results were obtained analyzing the data set restricted to locations with at least ten HPV infections in each age group (Web Table 4, Web Table 5).

**Table 2:** Effect of Time Elapsed Since Human Papillomavirus Detection (1993–2008) on Cervical Cancer Incidence (1993–2012) and Screening in Human Papillomavirus-Positive Women by Average Age of Sexual Debut in the Population, as Estimated Using Mixed Effect Linear Regression Models, PANDORA Model, Multiple Countries

	**Age 20–24 Years**				**Age 25–34 Years**			
**Average Age of Sexual Debut, years**	**CCI Rate** ^a^	**95% CI**	**% Increase in CCI** ^b^	**95% CI**	**CCI Rate** ^a^	**95% CI**	**% Increase in CCI** ^b^	**95% CI**
17	0.40	0.24, 0.68	19.37	17.26, 21.51	1.27	0.56, 2.89	4.98	3.59, 6.39
20	0.18	0.07, 0.43	29.77	24.81, 34.92	1.01	0.48, 2.10	8.32	7.12, 9.53
23	0.08	0.01, 0.44	41.08	30.16, 52.90	0.80	0.23, 2.79	11.76	9.23, 14.35
IRR (95% CI)^c^	0.56 (0.22, 1.39)				0.50 (0.14, 1.77)			
ICC	0.67				0.92			
	**Age 35–44 Years**				**Age 45–54 Years**			
	**CCI Rate** ^a^	**95% CI**	**% Increase in CCI** ^b^	**95% CI**	**CCI Rate** ^a^	**95% CI**	**% Increase in CCI** ^b^	**95% CI**
17	3.27	1.39, 7.67	0.36	−0.81, 1.55	5.43	2.22, 13.25	−1.76	−2.94, −0.56
20	2.74	1.33, 5.67	0.77	−0.09, 1.64	4.40	2.21, 8.76	−1.19	−1.92, −0.45
23	2.31	0.67, 7.99	1.18	−0.56, 2.94	3.56	1.09, 11.67	−0.61	−2.08, 0.88
IRR (95% CI)^c^	0.44 (0.12, 1.63)				0.47 (0.13, 1.72)			
ICC	0.94				0.95			

Abbreviations: CCI, cervical cancer incidence; CI, confidence interval; HPV, human papillomavirus; ICC, intraclass correlation coefficient; IRR, incidence rate ratio.

^a^ Per 1,000 HPV-positive women at HPV testing time: intercept of the model.

^b^ In HPV-positive women for every additional year elapsed since HPV testing: slope of the model accounting for the interaction of time elapsed between assessment of HPV prevalence and CCI assessment and average age of sexual debut in the population.

^c^ IRR due to screening; reference category is absence of screening.

**Figure 2:**
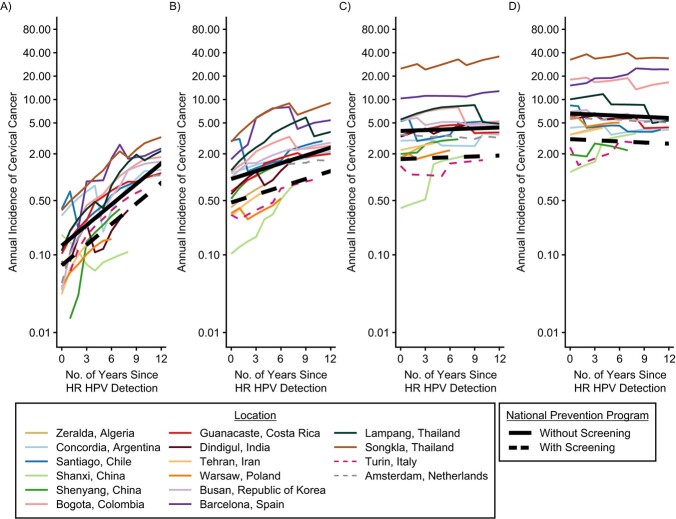
Cervical cancer incidence rates (1993–2012) in high-risk human papillomavirus (HR HPV)-positive women, PANDORA model, multiple countries. Results are shown by years since high-risk HPV detection (1993–2008), age group at HPV detection, screening implementation status and location. Model-based projections were drawn assuming the following average age at sexual debut 18.0 years (ages 20–24 years), 19.7 (ages 25–34 years), 20.1 (ages 35–44 years), and 20.5 (ages 45–54 years). Cervical cancer incidence rate is given per 100,000 woman-years. A) Ages 25–34 years; B) ages 35–44 years; C) ages 45–54 years; D) ages 55–64 years.

**Figure 3:**
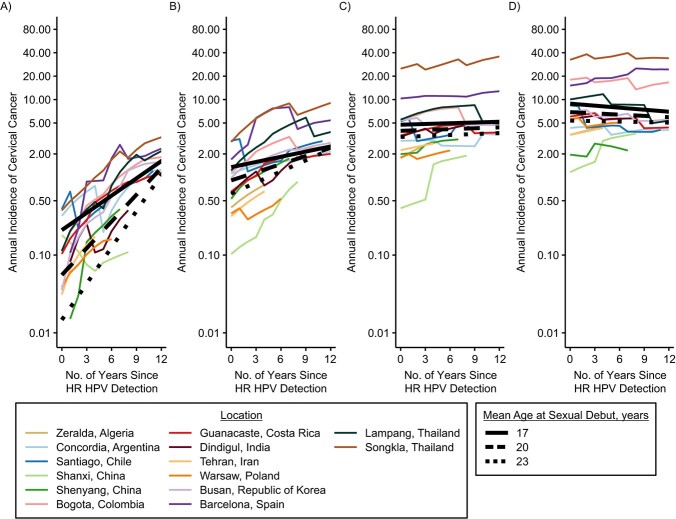
Cervical cancer incidence rates (1993–2012) in high-risk human papillomavirus (HR HPV)-positive women in locations without screening, PANDORA model, multiple countries. Results are shown by years since HR HPV detection (1993–2008), age–group at HR HPV detection, average age at sexual debut, and location. Cervical cancer incidence rate is given per 100,000 woman-years. A) Ages 25–34 years; B) ages 35–44 years; C) ages 45–54 years; D) ages 55–64 years.

The annual incidence of cervical cancer (per 1,000 HPV-positive women) over a period of 14 years following HPV detection, by age group at HPV detection, is shown in Figure 2A–D. Cervical cancer incidence is given for each of the 16 locations (thin curves), as well as the model-based predictions for locations with and without screening (thick lines). For 20- to 24-year-olds, cervical cancer incidence increased by approximately 1 order of magnitude over the 14-year period following high-risk HPV detection (e.g., 0.13 (90% PI: 0.06, 0.27) to 2.3 (90% PI: 1.1, 4.9) per 1,000 high-risk HPV infected women in locations without screening) (Web Table 6). With increasing age, increases in cervical cancer risk with time since high-risk HPV detection were less strong, so that among 45- to 54-year-olds, cervical cancer risk plateaued between 6.6 (90% PI: 4.0, 10.7) and 5.6 (90% PI: 3.4, 9.2) cancers per 1,000 high-risk HPV infected women over the 14 years (Web Table 6). Cervical cancer risk was higher, although not significantly, in locations without screening than in those with screening, and this difference in risk increased with age at HPV detection.

Estimated cervical cancer risk in the 14-year period following high-risk HPV detection, restricted to locations without screening, according to average age at sexual debut is shown in Figure 3A–D. Below age 35 years, average age at sexual debut was an important modifier of cervical cancer risk in HPV-positive women, with earlier sexual debut being associated with a higher initial risk. For example, at ages 20–24, the initial risk of cancer was 0.2 (90% PI: 0.09, 0.44), 0.06 (90% PI: 0.02, 0.13), and 0.02 (90% PI: 0.003, 0.06) for average sexual debut at ages 17, 20, and 23 years, respectively. However, these risks converge 14 years later: 2.4 (90% PI: 1.1, 5.2), 2.2 (90% PI: 0.9, 45.3), and 2 (90% PI: 0.5, 8.1), respectively. In contrast, in women with high-risk HPV detection at older ages, age at sexual debut was not a strong modifier of cervical cancer risk. More details about the estimated risk of cervical cancer in HPV-positive women for locations with and without screening are provided in Web Table 7.

To provide external validation of our estimates, we adapted our predictive model to reproduce the cervical cancer incidence measured in 2 cohort studies among high-risk HPV–positive and cytology-negative women. In one study, nested in a cervical cancer screening trial conducted in the Netherlands, estimated cervical cancer incidence was 1.9 (95% CI: 1.2, 3.2) per 1,000 women aged 29–61 years (19). In the second study, from Taiwan, estimated cervical cancer incidence was 3.7 per 1,000 women of 30–65 years of age (20). Of note, Taiwan implemented its nationwide screening program in 1995 and by 2005–2007 (i.e., at the end of the follow-up of the above-mentioned study), the coverage was about 50% (24).

According to our model-based estimates, cervical cancer incidence among high-risk HPV–positive women aged 30–59 years was 2.0 (90% PI: 0.7, 5.4) and 4.7 (90% PI: 3.0, 7.3) per 1,000 women in locations with screening and without screening, respectively (data not shown).

For Bhutan and Nigeria, our model-based estimates were capable of accurately predicting the incidence reported by the local cancer registry for the same birth cohorts (Table 3). For Georgia, our estimates were accurate for all age-groups, except for women 45–54 years in the target age group (corresponding to the “35–44” age group at time of HPV survey). For that age-group, because of low prevalence of high-risk HPV types, the model underestimated cervical cancer incidence. Last, cervical cancer incidence estimated with PANDORA and GLOBOCAN 2020 consistently overlapped for most age groups in all countries without high-quality cancer incidence data (Table 3). The lowest predicted incidence was in Pakistan, ranging from 0.4 (90% PI: 0.2, 0.9) to 13.7 (90% PI: 6.3, 29.5) per 100,000 women, among 20- to 24- and 50- to 59-year-old women, respectively, and the highest predicted incidence was in Guinea, ranging from 14.3 (90% PI: 6.7, 33.1) to 295.7 (90% PI: 123.2, 696.4) per 100,000 women.

## DISCUSSION

We have developed, parameterized, and validated PANDORA, a parsimonious model to predict age-specific cervical cancer incidence in a population in which we know age-specific high-risk HPV prevalence, average age at sexual debut, and screening implementation status. To do that, we characterized and quantified the relationship between age-specific HPV prevalence and cervical cancer incidence in the same female birth cohorts as a function of time lag between high-risk HPV prevalence and cancer incidence assessment. We found a strong positive effect of high-risk HPV prevalence on cervical cancer incidence, which increased with age at HPV detection and with time lag. This finding is consistent with the observation that prevalent HPV infections in older women are more likely to be persistent and have a higher risk of causing cervical cancer (11). In a mixed-model approach, we went on to show that annual cervical cancer incidence among women with prevalent HPV infection increased with age at HPV detection and, that in women below age 35, cervical cancer risk changed by average age at first sex and increased in the 14 years following high-risk HPV detection. In the absence of screening, depending on the combination of age at high-risk HPV detection and age at sexual debut, annual cervical cancer incidence ranged from 0.02 (90% PI: 0.003, 0.06) to 8.7 (90% PI: 3.5, 20.3) per 1,000 high-risk HPV–positive women. The model showed that annual cervical cancer incidence among HPV-positive women was lower (although not significantly) in screened than unscreened populations.

Our internal validation assessment suggests that model-based estimates are robust to data perturbation and fairly accurate to predict cervical cancer incidence in a new location. The external validity of our findings is supported by the comparison of our model-based estimate of cervical cancer incidence among HPV-positive women with empirical measurements from cohorts of HPV-positive and cytology-negative women from Amsterdam and Taiwan. PANDORA’s estimate for the screened population (i.e., 2.0 (90% PI: 0.7, 5.4) per 1,000 women), matches well data from Amsterdam (i.e., 1.9, 95% CI: 1.2, 3.2), where the organized screening program was fully implemented (19), whereas the estimate for unscreened populations (i.e., 4.7 (90% PI: 3.0, 7.3) per 1,000 women) was slightly higher than that observed in Taiwan (i.e., 3.7 per 1,000 women), where the population was partially screened (24). We also assessed the external validity by comparing the predicted cervical cancer incidence in Bhutan, Nigeria, and Georgia with age-specific incidence rates published through the local cancer registries that have not reached high enough quality to be considered in CI5. Therefore, as we have been able to predict annual cancer incidence from HPV prevalence in a set of countries in the IARC population-based HPV survey database without cancer registry data, we suggest that the PANDORA model could be used to draw predictions in other settings without cancer registration.

Ecologic inference has been previously used by Maucort-Boulch et al. (25) and Sharma et al. (26) to assess the correlation between HPV prevalence and cervical cancer incidence measured in the same time period and same age groups in 13 and 40 different locations worldwide, respectively. In both analyses, HPV prevalence was a better predictor of cervical cancer at older ages, probably because persistent infections are more likely to cause cervical cancer (11). This hypothesis is reinforced by our finding that the association between HPV prevalence and cervical cancer incidence increased not only with age at HPV detection but also with the time lag between high-risk HPV prevalence and cancer incidence measurement.

Our observations—1) that cervical cancer incidence among women below 35 years with prevalent high-risk HPV infection is modulated by age at first sex, as a proxy of early exposure to HPV infection, and 2) that cervical cancer incidence increases until approximately 45 years of age and subsequently remains constant—are consistent with data from unscreened populations (27) and with the hypothesis that risk inflection in middle age corresponds to a drop in circulating sex hormone levels during the perimenopausal period (28). Plummer et al. (29) also observed an increase in cervical cancer risk associated with time since first sexual intercourse, while analyzing a very large epidemiologic data set on cervical cancer. Similarly, their risk estimates flattened approximately at age 45 years and remained constant at older ages.

We have adapted our study design to account for some typical limitations of ecological studies. First, to account for the latency between exposure and outcome (which is missed by concurrent measurement of high-risk HPV prevalence and cervical cancer incidence), we assessed the 2 measurements in the same birth cohorts and incorporated the time lag between the 2 measurements. Second, we matched HPV and cervical cancer data from the same (or a similar) location, to geographically match, as far as possible, exposure and outcome measurements. Third, we accounted for age at first sex and cervical cancer screening as a possible confounder of the ecologic relationship in the same locations between HPV prevalence and cervical cancer incidence.

The effect of other location-related determinants of cervical cancer risk given high-risk HPV detection, such as human immunodeficiency virus status, reproductive behavior factors, hormonal contraceptive use, and smoking, for which we were unable to explicitly account, may also have been captured by the between-location variance. Finally, high-risk HPV prevalence survey data and incidence data from the cancer registries for the same geographic areas were not available for 4 out of 16 locations. Hence, for these settings, we selected cancer registries with comparable characteristics, whenever possible in the same region or country (see Web Table 1 for more details).

Consequently, our predictions of the annual incidence of cervical cancer in countries not represented in CI5 are based on average age at sexual debut, time elapsed since HPV assessment, and the age-specific high-risk HPV prevalence assessed in each survey. The accuracy of the reported predictions relies on the assumption that the age-specific high-risk HPV prevalence assessed in the survey is representative of prevalence in the whole country. Our estimates of the risk of cervical cancer among high-risk HPV–positive women are based upon the systematic use of the GP5+/6+ test, which is validated for clinical purposes. Hence our findings should be applicable to other population-based data sets obtained using HPV tests with a similar sensitivity but might be less applicable to prevalence data generated with more sensitive methods.

**Table 3:** Expected and Observed Annual Number and Incidence of Cervical Cancer Cases per 100,000 Women (1997–2014), 5 Years After the Implementation of the Surveys^a^, PANDORA Model, Multiple Countries

**HPV Survey Characteristic**			**Country-Level Population and Expected High-Risk HPV–Positive Women** ^**b**^		**Projection** ^**c**^						
**AgeGroup,years**	**High-Risk HPV Prevalence, %**	**Average Age at Sexual Debut, years**	**No. of Women**	**No. of HPV Positive Women**	**Target Age Group, years**	**No. of Expected Cancers**	**90%PI**	**Incidence per 100,000 Women**	**90% PI**	**Observed Incidence per100,000Women** ^**d**^	**95% CI**
*Thimphu and Lungthenphu, Bhutan, 2011–2012*											
20–24	22.3	19	32,076	7,137	25–29	2	1, 4	6.0	2.8, 13.3	2.9	1.4, 5.3
25–34	18.9	20	48,529	9,170	30–39	12	7, 21	25.1	14.1, 44.3	22.1	16.9, 28.5
35–44	14.3	20	33,459	4,769	40–49	20	12, 34	59.8	36.4, 100.8	54.7	44.3, 66.8
45–54	11.2	19	21,116	2,373	50–59	17	9, 28	78.5	44.7, 132.1	56.7	44.2, 71.6
*Ibadan, Nigeria, 1999*											
20–24	19.0	17	5,557,934	1,058,654	30–34	1,163	566, 2,696	20.9	10.2, 48.5	4.7	2.2, 8.6
25–34	14.3	19	8,182,426	1,168,918	35–44	2,513	1,414, 4,460	30.7	17.3, 54.5	23.9	18.6, 30.1
35–44	18.7	19	5,638,379	1,051,936	45–54	4,678	2,721, 8,160	83.0	48.3, 144.7	70.1	58.8, 82.9
45–54	16.8	20	3,978,221	669,803	55–64	4,213	2,472, 6,950	105.9	62.1, 174.7	95.9	78.9, 115.5
*Tbilisi, Georgia, 2007*											
20–24	12.3	19	175,471	21,509	30–34	19	8, 40	10.7	4.9, 22.8	8.9	6.5, 11.8
25–34	12.2	20	321,833	39,408	35–44	80	45, 139	24.9	14.0, 43.2	23.0	20.1, 26.2
35–44	4.8	22	335,425	15,973	45–54	61	35, 112	18.3	10.5, 33.4	39.9	36.1, 44.0
45–54	6.2	23	317,977	19,669	55–64	103	54, 195	32.3	16.9, 61.2	32.3	18.9, 35.9
*Conakry, Guinea, 2006*											
20–24	29.9	17	422,643	126,398	25–29	61	28, 140	14.3	6.7, 33.1	11.7	N/A
25–34	28.7	17	633,390	181,806	30–39	313	153, 667	49.4	24.2, 105.3	35.6	N/A
35–44	25.4	17	447,604	113,864	40–49	545	257, 1,180	121.7	57.3, 263.5	156.1	N/A
45–54	36.0	17	340,426	122,455	50–59	1,007	420, 2,371	295.7	123.2, 696.4	145.9	N/A
*Ulanbataar, Mongolia, 2005*											
20–24	40.2	19	129,903	52,260	25–29	13	6, 26	9.8	4.6, 20.4	5.9	N/A
25–34	24.2	21	223,403	54,132	30–39	70	40, 119	31.2	17.8, 53.4	19.2	N/A
35–44	17.5	21	181,801	31,798	40–49	123	75, 211	67.4	41.4, 115.9	79.7	N/A
45–54	17.3	20	106,259	18,354	50–59	115	70, 184	108.2	66.2, 173	59.0	N/A
*Bharatpur, Nepal, 2006–2007*											
20–24	5.7	17	1,158,460	65,728	25–29	34	15, 77	3.0	1.3, 6.6	0.3	N/A
25–34	5.6	18	1,920,287	107,384	30–39	178	91, 336	9.3	4.8, 17.5	6.1	N/A
35–44	7.9	18	1,335,815	106,094	40–49	511	252, 1,011	38.2	18.9, 75.6	43.6	N/A
45–54	5.5	17	944,642	51,761	50–59	418	173, 1,021	44.3	18.4, 108	44.7	N/A
*South Karachi, Pakistan, 2006*											
20–24	1.2	18	7,090,156	85,424	25–29	29	14, 61	0.4	0.2, 0.9	0.8	N/A
25–34	2.1	20	10,730,124	224,480	30–39	306	175, 534	2.8	1.6, 5.0	4.9	N/A
35–44	0.8	19	7,984,107	64,649	40–49	284	165, 507	3.6	2.1, 6.4	23.4	N/A
45–54	1.7	17	5,244,948	91,482	50–59	719	329, 1,547	13.7	6.3, 29.5	20.8	N/A
*Kigali, Rwanda, 2013–2014*											
20–24	28.4	18	515,301	146,404	25–29	56	28, 117	10.9	5.5, 22.7	5.9	N/A
25–34	21.2	20	1,017,610	215,678	30–39	302	177, 518	29.7	17.4, 50.9	22.5	N/A
35–44	14.3	21	589,561	84,223	40–49	341	211, 571	57.8	35.8, 96.8	101.9	N/A
45–54	13.0	21	376,934	49,061	50–59	304	185, 486	80.7	49.2, 128.8	93.1	N/A
*Port Vila, Vanuatu, 2009–2010*											
20–24	31.6	18	11,876	3,750	25–29	1	1, 3	12.0	5.5, 25.6	15.2	N/A
25–34	21.1	19	17,714	3,746	30–39	6	3, 10	32.6	18.5, 56.7	33.1	N/A
35–44	18.0	19	12,896	2,327	40–49	10	6, 19	80.3	45.4, 150.2	87.1	N/A
45–54	9.3	19	9,452	877	50–59	6	3, 11	64.7	36.4, 114.4	29.9	N/A
*Ho Chi Minh City, Viet Nam, 1997*											
20–24	9.4	20	3,549,293	334,051	25–29	75	33, 163	2.1	0.9, 4.6	2.1	N/A
25–34	5.8	23	6,123,615	356,024	30–39	385	181, 806	6.3	3.0, 13.2	6.1	N/A
35–44	7.0	24	4,465,621	313,800	40–49	1,055	450, 2,583	23.6	10.1, 57.8	25.4	N/A
45–54	4.5	23	2,299,986	103,870	50–59	530	254, 1,081	23.0	11.0, 47.0	18.8	N/A

Abbreviations: CI, confidence interval; HPV, high–risk human papillomavirus; IARC, International Agency for Research on Cancer; N/A, not applicable; PI, prediction interval.

^a^ See Web Table 8 for IARC population-based survey references.

^b^ Source: United Nations Department of Economic and Social Affairs. Population Dynamics. World Population Prospects 2019 (30).

^c^ For Nigeria and Georgia, projections were obtained for the population of the corresponding country 10 years after the implementation of the surveys to match the age range reported in the cancer registry; for all other countries, projections were obtained for the population of the corresponding country 5 years after the implementation of the surveys.

^d^ For Bhutan, Nigeria, and Georgia, the observed cervical cancer incidence in 2014–2018 was obtained from population-based cancer registries; for all other countries it was obtained from GLOBOCAN 2020 (https://gco.iarc.fr/).

In conclusion, using worldwide, high-quality, standardized data on age-specific HPV prevalence and cervical cancer incidence from the same location, we have built a model to estimate age-specific cervical cancer incidence over time elapsed since high-risk HPV detection, as a function of average age at first sex in the female population. PANDORA could be particularly useful in designing and planning cancer control programs in settings without cancer registries as a tool to predict the expected burden of cervical cancer from high-quality data collected through population-based cross-sectional HPV prevalence surveys, which are relatively easy to implement.

## Supplementary Material

Web_Material_kwab254
