# Correction to “Predicting cohort-specific cervical cancer incidence from population-based surveys of human papilloma virus prevalence: a worldwide study”

**DOI:** 10.1093/aje/kwae310

**Published:** 2026-02-27

**Authors:** Rosa Schulte-Frohlinde, Damien Georges, Gary Clifford, Iacopo Baussano

In the article “Predicting Cohort-Specific Cervical Cancer Incidence from Population-Based Surveys of Human Papilloma Virus Prevalence: A Worldwide Study” by Schulte-Frohlinde et al.,^1^ there was an error in the online and print publications. The legends for Figures 2 (page 408) and 3 (page 409) incorrectly gave the cervical cancer incidence rate to be “per 100,000 woman-years” for age ranges “A) Ages 25–34 years; B) ages 35–44 years; C) ages 45–54 years; D) ages 55–64 years.” This should be corrected to cervical cancer incidence rate is given “per 1,000 HR HPV-positive woman-years” for age ranges “A) Ages 20–24 years; B) ages 25–34 years; C) ages 35–44 years; D) ages 45–54 years.”

The authors regret these errors.
