# Supplementary material for: Predicting Cohort-Specific Cervical Cancer Incidence From Population-Based Surveys of Human Papilloma Virus Prevalence: A Worldwide Study
Source: Am J Epidemiol. 2021 Oct 15;191(3):402–12. doi: 10.1093/aje/kwab254 (PMC8895389; doi:10.1093/aje/kwab254)
Supplement: Web_Material_kwab254 [file web_material_kwab254.pdf]

Web Material

Predicting Cohort-Specific Cervical Cancer Incidence From Population-Based Surveys of Human Papilloma Virus Prevalence: A Worldwide Study

Rosa Schulte-Frohlinde, Damien Georges, Gary M. Clifford, and Iacopo Baussano

Table of Contents

Web Appendix 2
Web Table 1 3
Web Table 2 4
Web Table 3 5
Web Table 4 6
Web Table 5 7
Web Table 6 8
Web Table 7 9
Web Table 8 11
Web Figure 1 12
References 13

Web Appendix. PANDORA Model - formulae

Pseudo-R2 for correlation model

To assess goodness of fit of the correlation model, we used a pseudo-R2 index (see Kraus BJ et al. Neuron. 2015 doi:10.1016/j.neuron.2015.09.031, for further details). This index is based on model log-likelihood and assessment of the proportion of the variance captured by our model.

$$pR_M^2 = \frac{ln(\Gamma_M) - ln(\Gamma_{Null})}{ln(\Gamma_{Sat}) - ln(\Gamma_{Null})}$$

where pR2M is the pseudo-R2 for a model of interest, ln(ΓM) is the log-likelihood of the model of interest, ln(ΓNull) is the log-likelihood of the null model (constant cervical cancer incidence rate) and ln(ΓSat) is the log-likelihood of the saturated model (each combination of location, women's age group, and time lag has its own cervical cancer incidence rate). Pseudo-R2 has been computed for each combination of time lag category between human papillomavirus (HPV) prevalence survey and cervical cancer incidence rate from cancer registry data collection.

Model to estimate cervical cancer incidence rate among high-risk human papillomavirus-positive women

To infer the cervical cancer incidence rate in high-risk (HR) HPV-positive women in a given age group we used a linear mixed-effect model as specified below:

$$log\left(CCIR_{ij}\right) \sim \beta_0 + b_{0i} + \beta_1 YSD_j + \beta_2 ASD_i + \beta_3 YSD_j . AASD_i + \beta_4 NSP_i + \epsilon_{ij}$$

where CCIRij is the cervical cancer incidence rate for the i-th country at time j, YSD is the number of years elapsed since HPV detection at time j, ASDi is the average age at sexual debut for the i-th country, NSPi is the existence of a national cervical cancer screening program for the i-th country, b0i is the random intercept for the i-th county, β0, β1, β2, β3 and β4 are the overall fixed effects, and εij is the random error.

Prediction of cervical cancer incidence

Predictions were obtained by drawing from the above-mentioned model a sampling distribution for the fixed effects and estimating the fitted values across the distribution. The median of the fitted value was reported as central estimate; quantiles 5% and 95% were reported as prediction interval boundaries. This procedure has been conducted using predictInterval function from MerTools package (v 0.5.2).
(https://cran.r-project.org/web/packages/merTools/index.html)

Web Table 1. PANDORA Model **-** sources of available data for each location.

	Human papillomavirus Cross-sectional survey		Cancer incidence data		
**Country**	**Location**	**Year of the survey**	**Source**	**Cancer registry**	**With/without screening**
Algeria	Zeralda	2007–2008	CI5, Volume X-XI	Setif	Without
Argentina	Concordia	1998	CI5, Volume IX-XI	Bahia Blanca/Entre Rios[a]	Without
Chile	Santiago	2001–2003	CI5, Volume IX-XI	Antofagasta/Valdivia[b]	Without
China	Shanxi	2004	CI5, Volume X-XI	Cixian	Without
China	Shenyang	2005	CI5, Volume X-XI	Shenyang/Harbin[c]	Without
Colombia	Bogota	1993–1995	CI5, Volume VIII-XI	Cali	Without
Costa Rica	Guanacaste	1993–1994	CI5, Volume VIII-XI	Costarica	Without
India	Dindigul	2004	CI5, Volume X-XI	Ambilikkai	Without
Iran	Tehran	2008	CI5, Volume X-XI	Gholestan	Without
Italy	Turin	2002	CI5, Volume IX-XI	Turin	With
Netherlands	Amsterdam	1995–1998	CI5, Volume IX-XI	The Netherlands	With
Poland	Warsaw	2006	CI5, Volume X-XI	Kielce	Without
Republic of Korea	Busan	1999–2000	CI5, Volume IX-XI	Busan	Without
Spain	Barcelona	1998–2000	CI5, Volume IX-XI	Tarragona	Without
Thailand	Lampang	1997–1998	CI5, Volume IX-XI	Lampang	Without
Thailand	Songkla	1999–2000	CI5, Volume IX-XI	Songkla	Without
Viet Nam	Ho Chi Minh City	1997	CI5, Volume XI	Ho Chi Minh	Without

[a] Data from Bahia Blanca and Entre Rios cancer registry cover 1998–2007 and 2008–12 period, respectively.
[b] Data from Antofagasta and Valdivia cancer registry cover 2001–2002 and 2003–12 period, respectively.
[c] Data from Shenyang and Harbin cancer registry cover 2005–2008 and 2009–12 period, respectively.

Note: For 12 locations, high-risk human papillomavirus prevalence survey data and incidence data from the cancer registries were available for the same geographic areas. For the prevalence studies in Warsaw, Poland; Barcelona, Spain; Bogota, Colombia; Shanxi, China; and Tehran, Iran, we selected cancer registries, with comparable characteristics whenever possible, in the same region or country, namely: Kielce, showing similarly low rates as Warsaw in previous years; Tarragona, the next largest city after Barcelona in the region of Catalonia; Cali, the largest Colombian cancer registry; Cixian, as the study in Shanxi also included women from this region; and Gholestan province in Iran.

Web Table 2. PANDORA Model **-** average at sexual debut, by age at human papillomavirus detection (1993–2008) and location.

**Location**	**Mean age at sexual debut, years**			
	**20–24**	**25–34**	**35–44**	**45–54**
Algeria, Zeralda	19.8	22.3	22.6	21.5
Argentina, Concordia	17	17.8	18.3	19.9
Chile, Santiago	16.7	17.6	18.7	19.7
China, Shanxi	19.4	20.5	20.3	19.4
China, Shenyang	20.3	22.5	22.9	24.7
Colombia, Bogota	17.2	19	19.7	19.5
Costa Rica, Guanacaste	16.8	18.3	18.2	18.9
India, Dindigul	17.8	17.9	18.1	17.7
Iran, Tehran	18.7	20.7	20.8	20.5
Italy, Turin	17.1	18.8	18.7	19.2
Netherlands, Amsterdam	16.7	16.7	16.7	16.7
Poland, Warsaw	18.5	19.4	20	20.5
Republic of Korea, Busan	20.7	23.9	24.3	23.1
Spain, Barcelona	17.9	19.6	19.7	22.1
Thailand, Lampang	18.1	20.2	20.7	20.6
Thailand, Songkla	17.8	19.6	20.1	20.3

Web Table 3. PANDORA Model - estimated increase in cervical cancer incidence per 10,000 women-year per 1% increase in high-risk human papillomavirus prevalence by age at human papillomavirus detection and time lag between human papillomavirus prevalence (1993–2008) and cancer incidence (2008–2012) measurement, restricted to 15 locations without screening.

Age Group, years<br>Time Lag, years	Intercept	95% CI	Estimated Increase	95% CI	Pseudo-R^2
25–34					
<10	0	0, 1.5	0.9	0.7, 1.1	0.87
≥10	12.7	9.9, 15.7	1	0.7, 1.3	0.61
35–44					
<10	0.7	0, 3.4	2.5	2.2, 2.8	0.57
≥10	26.7	23.2, 30.3	0.7	0, 1.3	0.43
45–54					
<10	4.5	0, 10.0	3.0	2.5, 3.6	0.56
≥10	34.5	29.8, 39.2	0	-0.8, 0.9	0.31
55–64					
<10	0	0, 6.8	5.0	3.9, 6.2	0.54
≥10	30.8	24.7, 36.8	1.5	0.4, 2.6	0.56

Abbreviations: CI, confidence interval.

Web Table 4. PANDORA Model - R^2 for internal cross-validation procedures, by location.

	All locations		Locations with more than 10 high-risk human papillomaviruses	
**Age group, years**	**Random procedure[a]**	**Blocked procedure[b]**	**Random procedure[a]**	**Blocked procedure[b]**
20–24	0.84	0.79	0.85	0.79
25–34	0.90	0.73	0.90	0.76
35–44	0.92	0.58	0.83	0.56
45–54	0.93	0.59	0.86	0.61
Average	0.90	0.67	0.86	0.68

[a] The dataset was split into ten parts, nine parts being used to train the model, while the remaining part of the dataset was used to evaluate the model, and this operation was repeated ten times.
[b] We trained the model leaving out one country at a time and predicted cervical cancer incidence for that country, and this operation was repeated for every single country.

Web Table 5. PANDORA Model - sensitivity analysis, effect (95% confidence intervals) of time elapsed since human papillomavirus detection (1993–2008) on cervical cancer incidence (1993–2012) and screening among in human papillomavirus-positive women by average age of sexual debut in the population, as estimated using mixed effect linear regression models. The analysis is restricted to surveys with at least 10 high-risk human papillomavirus infections in each age group.

Average Age of Sexual Debut in the population, years	Age 20–24 years				Age 25–34 years			
	CCI Rate[a]	95% CI	% Increase in CCI[b]	95% CI	CCI Rate[a]	95% CI	% Increase in CCI[b]	95% CI
17	0.36	0.23, 0.55	19.4	17.19, 21.65	1.09	0.55, 2.18	5.04	3.71, 6.38
20	0.18	0.09, 0.37	29.83	24.71, 35.17	0.92	0.5, 1.71	8.41	7.19, 9.64
23	0.09	0.02, 0.37	41.18	30.05, 53.26	0.78	0.28, 2.21	11.89	9.4, 14.44
IRR (95% CI) due to screening[c]	0.7 (0.33, 1.46)				0.65 (0.23, 1.83)			
ICC	0.56				0.89			
	Age 35–44 years				Age 45–54 years			
17	2.76	1.52, 5.01	0.26	-1, 1.55	5.29	2.89, 9.69	-1.47	-2.68, -0.25
20	2.48	1.5, 4.1	0.56	-0.44, 1.57	3.72	2.32, 5.96	-1.9	-2.72, -1.07
23	2.23	0.95, 5.26	0.85	-1.08, 2.82	2.61	1.15, 5.96	-2.32	-3.99, -0.63
IRR (95% CI) due to screening[c]	0.59 (0.24, 1.45)				0.53 (0.23, 1.26)			
ICC	0.86				0.88			

Abbreviations: CCI, cervical cancer incidence; CI, confidence interval; ICC, intraclass correlation coefficient; IRR, incidence rate ratio.

[a] Per 1,000 human papillomavirus (HPV)-positive women at HPV testing time: intercept of the model.

[b] In HPV-positive women for every additional year elapsed since HPV testing: slope of the model accounting for the interaction of time elapsed between assessment of HPV prevalence and CCI assessment and average age of sexual debut in the population, years.

[c] Reference category is absence of screening.

Web Table 6. PANDORA Model - detailed estimates of cervical cancer risk (90% prediction intervals) in 1,000 high-risk human papillomavirus-positive women by age group at human papillomavirus detection (1993–2008), time lag between high-risk human papillomavirus and cervical cancer incidence measurement (1993–2012), and screening implementation status.

Time Lag, years[a]	Locations without screening				Locations with screening			
	**20–24 years**	**25–34 years**	**35–44 years**	**45–54 years**	**20–24 years**	**25–34 years**	**35–44 years**	**45–54 years**
0	0.13 (0.06, 0.27)	0.95 (0.55, 1.65)	3.95 (2.36, 6.64)	6.56 (4.01, 10.68)	0.12 (0.04, 0.31)	0.62 (0.23, 1.77)	2.13 (0.75, 6)	3.9 (1.38, 10.81)
1	0.15 (0.07, 0.33)	1.02 (0.58, 1.76)	3.99 (2.41, 6.67)	6.51 (3.96, 10.54)	0.14 (0.05, 0.38)	0.66 (0.24, 1.91)	2.14 (0.74, 6.04)	3.87 (1.38, 10.61)
2	0.19 (0.09, 0.41)	1.1 (0.64, 1.91)	4 (2.4, 6.74)	6.44 (3.93, 10.31)	0.16 (0.06, 0.44)	0.69 (0.26, 1.99)	2.14 (0.74, 6.11)	3.78 (1.36, 10.45)
3	0.24 (0.11, 0.49)	1.19 (0.69, 2.07)	4.05 (2.44, 6.79)	6.35 (3.89, 10.21)	0.2 (0.07, 0.52)	0.73 (0.27, 2.11)	2.15 (0.75, 6.02)	3.73 (1.32, 10.27)
4	0.29 (0.14, 0.61)	1.29 (0.75, 2.24)	4.08 (2.47, 6.82)	6.26 (3.8, 10.16)	0.23 (0.09, 0.63)	0.78 (0.29, 2.24)	2.17 (0.76, 6.17)	3.69 (1.32, 10.12)
5	0.35 (0.17, 0.75)	1.39 (0.81, 2.41)	4.08 (2.47, 6.83)	6.2 (3.8, 10.06)	0.28 (0.1, 0.75)	0.82 (0.3, 2.32)	2.17 (0.75, 6.22)	3.61 (1.28, 9.82)
6	0.44 (0.21, 0.93)	1.51 (0.87, 2.59)	4.13 (2.5, 6.88)	6.15 (3.73, 9.97)	0.33 (0.12, 0.89)	0.86 (0.32, 2.49)	2.18 (0.77, 6.2)	3.56 (1.28, 9.81)
7	0.54 (0.26, 1.13)	1.62 (0.94, 2.78)	4.17 (2.51, 6.92)	6.11 (3.68, 9.82)	0.39 (0.15, 1.06)	0.92 (0.34, 2.64)	2.2 (0.76, 6.24)	3.52 (1.25, 9.49)
8	0.67 (0.32, 1.42)	1.75 (1.02, 3.02)	4.22 (2.53, 7.02)	6.01 (3.65, 9.73)	0.47 (0.17, 1.26)	0.97 (0.36, 2.77)	2.18 (0.77, 6.1)	3.41 (1.23, 9.38)
9	0.81 (0.39, 1.71)	1.89 (1.1, 3.26)	4.22 (2.56, 7.06)	5.93 (3.65, 9.55)	0.56 (0.21, 1.51)	1.02 (0.38, 2.95)	2.2 (0.78, 6.23)	3.37 (1.2, 9.27)
10	1 (0.48, 2.1)	2.06 (1.18, 3.52)	4.26 (2.55, 7.11)	5.87 (3.58, 9.55)	0.67 (0.24, 1.79)	1.08 (0.4, 3.1)	2.22 (0.78, 6.32)	3.34 (1.19, 9.09)
11	1.24 (0.59, 2.63)	2.21 (1.28, 3.8)	4.3 (2.61, 7.19)	5.79 (3.53, 9.45)	0.8 (0.29, 2.13)	1.15 (0.42, 3.28)	2.22 (0.78, 6.33)	3.27 (1.17, 8.86)
12	1.52 (0.72, 3.24)	2.39 (1.38, 4.12)	4.34 (2.62, 7.25)	5.72 (3.5, 9.25)	0.95 (0.35, 2.56)	1.21 (0.45, 3.51)	2.23 (0.77, 6.37)	3.22 (1.16, 8.89)
13	1.87 (0.86, 4.04)	2.58 (1.49, 4.45)	4.36 (2.62, 7.29)	5.67 (3.44, 9.28)	1.12 (0.41, 3.06)	1.28 (0.47, 3.6)	2.24 (0.78, 6.43)	3.16 (1.13, 8.62)
14	2.32 (1.08, 4.9)	2.79 (1.6, 4.85)	4.41 (2.64, 7.39)	5.6 (3.39, 9.23)	1.34 (0.48, 3.55)	1.35 (0.5, 3.9)	2.26 (0.79, 6.43)	3.11 (1.12, 8.5)

Abbreviations: HR HPV, high-risk human papillomavirus.
Model-based projections were drawn assuming the following average age at sexual debut in locations without and with screening, respectively, 18.3 and 16.9 years (age group 20–24); 19.9 and 17.8 years (age group 25–34); 20.3 and 17.7 (age group 35–44); and 20.6 and 17.9 (age group 45–54).
[a] Years elapsed between HR HPV detection and cervical cancer incidence assessment.

Web Table 7. PANDORA Model - detailed estimates of cervical cancer risk (90% prediction intervals) in 1,000 high-risk human papillomavirus-positive women by age group at human papillomavirus detection (1993–2008), time lag between high-risk human papillomavirus and cervical cancer incidence measurement (1993–2012), average age at sexual debut, and screening implementation status.

Time Lag, years[a]	Age Sexual Debut, years	Without screening				With screening			
		20–24 years	25–34 years	35–44 years	45–54 years	20–24 years	25–34 years	35–44 years	45–54 years
0	17	0.2 (0.09, 0.44)	1.36 (0.63, 3)	4.92 (2.1, 11.33)	8.67 (3.49, 20.28)	0.11 (0.04, 0.29)	0.67 (0.23, 1.87)	2.12 (0.76, 6.28)	4.08 (1.48, 11.75)
0	20	0.06 (0.02, 0.13)	0.91 (0.53, 1.61)	4 (2.39, 6.74)	6.88 (4.18, 11.25)	0.03 (0.01, 0.1)	0.45 (0.15, 1.43)	1.75 (0.53, 5.55)	3.28 (1.09, 9.94)
0	23	0.02 (0, 0.06)	0.62 (0.27, 1.38)	3.27 (1.6, 6.66)	5.43 (2.82, 10.84)	0.01 (0, 0.05)	0.31 (0.07, 1.34)	1.44 (0.31, 6.34)	2.56 (0.63, 11.05)
1	17	0.24 (0.11, 0.53)	1.42 (0.65, 3.14)	4.93 (2.13, 11.3)	8.58 (3.42, 19.96)	0.13 (0.05, 0.35)	0.71 (0.25, 1.97)	2.12 (0.76, 6.22)	4 (1.45, 11.51)
1	20	0.07 (0.03, 0.17)	0.99 (0.57, 1.73)	4.03 (2.41, 6.68)	6.79 (4.14, 11.1)	0.04 (0.01, 0.13)	0.49 (0.16, 1.54)	1.76 (0.54, 5.53)	3.23 (1.07, 9.66)
1	23	0.02 (0.01, 0.08)	0.69 (0.31, 1.55)	3.31 (1.63, 6.69)	5.4 (2.8, 10.72)	0.01 (0, 0.07)	0.34 (0.08, 1.46)	1.44 (0.31, 6.49)	2.54 (0.63, 11.02)
2	17	0.29 (0.13, 0.62)	1.48 (0.69, 3.27)	4.95 (2.1, 11.45)	8.41 (3.36, 19.58)	0.16 (0.06, 0.41)	0.74 (0.26, 2.06)	2.13 (0.77, 6.29)	3.96 (1.43, 11.38)
2	20	0.1 (0.04, 0.22)	1.07 (0.62, 1.87)	4.04 (2.42, 6.77)	6.69 (4.07, 10.96)	0.05 (0.02, 0.17)	0.54 (0.17, 1.66)	1.78 (0.54, 5.63)	3.2 (1.05, 9.61)
2	23	0.03 (0.01, 0.12)	0.78 (0.35, 1.73)	3.35 (1.65, 6.83)	5.39 (2.75, 10.61)	0.02 (0, 0.09)	0.39 (0.09, 1.64)	1.47 (0.32, 6.53)	2.53 (0.62, 10.86)
3	17	0.34 (0.16, 0.74)	1.56 (0.73, 3.47)	4.97 (2.12, 11.33)	8.26 (3.32, 19.22)	0.19 (0.07, 0.5)	0.78 (0.27, 2.18)	2.14 (0.77, 6.3)	3.89 (1.42, 11.12)
3	20	0.12 (0.05, 0.28)	1.16 (0.67, 2.02)	4.1 (2.44, 6.84)	6.61 (4.05, 10.86)	0.07 (0.02, 0.22)	0.58 (0.19, 1.77)	1.8 (0.55, 5.71)	3.16 (1.05, 9.46)
3	23	0.04 (0.01, 0.17)	0.87 (0.38, 1.93)	3.4 (1.67, 6.9)	5.35 (2.74, 10.69)	0.03 (0, 0.13)	0.43 (0.1, 1.87)	1.49 (0.32, 6.59)	2.52 (0.62, 10.73)
4	17	0.41 (0.18, 0.9)	1.63 (0.77, 3.6)	4.93 (2.13, 11.42)	8.13 (3.27, 18.79)	0.23 (0.09, 0.6)	0.81 (0.29, 2.3)	2.16 (0.77, 6.33)	3.81 (1.38, 10.86)
4	20	0.16 (0.07, 0.37)	1.26 (0.74, 2.2)	4.11 (2.47, 6.85)	6.56 (4.02, 10.7)	0.09 (0.03, 0.29)	0.63 (0.2, 1.93)	1.8 (0.55, 5.71)	3.12 (1.03, 9.53)
4	23	0.06 (0.02, 0.23)	0.97 (0.43, 2.15)	3.43 (1.68, 7)	5.28 (2.72, 10.51)	0.04 (0.01, 0.19)	0.48 (0.11, 2.09)	1.49 (0.33, 6.67)	2.49 (0.61, 10.82)
5	17	0.49 (0.22, 1.06)	1.71 (0.79, 3.78)	4.96 (2.13, 11.55)	7.95 (3.17, 18.45)	0.27 (0.1, 0.71)	0.85 (0.3, 2.42)	2.15 (0.78, 6.3)	3.73 (1.36, 10.59)
5	20	0.21 (0.09, 0.48)	1.36 (0.79, 2.39)	4.13 (2.47, 6.91)	6.45 (3.93, 10.48)	0.12 (0.03, 0.38)	0.68 (0.22, 2.1)	1.81 (0.56, 5.77)	3.06 (1.01, 9.25)
5	23	0.09 (0.02, 0.33)	1.09 (0.48, 2.42)	3.46 (1.7, 7.05)	5.27 (2.7, 10.35)	0.05 (0.01, 0.26)	0.54 (0.12, 2.32)	1.53 (0.33, 6.84)	2.46 (0.61, 10.66)
6	17	0.57 (0.26, 1.26)	1.8 (0.84, 4.02)	5 (2.12, 11.43)	7.83 (3.11, 18.2)	0.33 (0.12, 0.85)	0.9 (0.31, 2.53)	2.16 (0.77, 6.41)	3.67 (1.32, 10.6)
6	20	0.27 (0.11, 0.63)	1.48 (0.86, 2.58)	4.17 (2.5, 6.96)	6.36 (3.88, 10.42)	0.15 (0.04, 0.48)	0.74 (0.24, 2.29)	1.83 (0.55, 5.76)	3.05 (1.02, 9.29)
6	23	0.13 (0.03, 0.47)	1.21 (0.54, 2.71)	3.53 (1.71, 7.11)	5.23 (2.7, 10.34)	0.07 (0.01, 0.36)	0.6 (0.14, 2.61)	1.53 (0.33, 6.76)	2.46 (0.6, 10.56)
7	17	0.69 (0.31, 1.5)	1.89 (0.88, 4.14)	5 (2.17, 11.64)	7.72 (3.05, 17.75)	0.39 (0.14, 1.02)	0.94 (0.33, 2.68)	2.17 (0.77, 6.38)	3.6 (1.33, 10.34)
7	20	0.35 (0.15, 0.81)	1.61 (0.93, 2.81)	4.22 (2.52, 7.02)	6.32 (3.82, 10.27)	0.2 (0.06, 0.63)	0.8 (0.26, 2.49)	1.84 (0.56, 5.83)	2.99 (0.99, 9.01)
7	23	0.18 (0.05, 0.65)	1.36 (0.6, 3.02)	3.56 (1.73, 7.18)	5.23 (2.68, 10.38)	0.1 (0.02, 0.52)	0.67 (0.16, 2.93)	1.55 (0.34, 6.86)	2.44 (0.6, 10.58)
8	17	0.83 (0.38, 1.82)	1.99 (0.93, 4.41)	5.01 (2.14, 11.59)	7.56 (3, 17.59)	0.46 (0.17, 1.18)	0.99 (0.35, 2.78)	2.17 (0.77, 6.44)	3.53 (1.28, 10.23)
8	20	0.45 (0.19, 1.04)	1.74 (1.01, 3.02)	4.25 (2.54, 7.12)	6.23 (3.8, 10.18)	0.25 (0.08, 0.82)	0.86 (0.28, 2.71)	1.86 (0.57, 5.91)	2.96 (0.97, 8.87)

Time Lag, years[a]	Age Sexual Debut, years	Without screening				With screening			
		20–24 years	25–34 years	35–44 years	45–54 years	20–24 years	25–34 years	35–44 years	45–54 years
8	23	0.25 (0.06, 0.92)	1.52 (0.67, 3.37)	3.61 (1.74, 7.25)	5.16 (2.66, 10.33)	0.14 (0.03, 0.73)	0.75 (0.17, 3.3)	1.57 (0.34, 6.95)	2.42 (0.6, 10.52)
9	17	0.98 (0.45, 2.13)	2.07 (0.96, 4.63)	5.04 (2.15, 11.45)	7.4 (2.94, 17.21)	0.54 (0.21, 1.46)	1.04 (0.37, 2.94)	2.19 (0.79, 6.44)	3.49 (1.28, 10.02)
9	20	0.59 (0.25, 1.37)	1.89 (1.08, 3.28)	4.28 (2.56, 7.2)	6.16 (3.75, 10.04)	0.33 (0.1, 1.07)	0.94 (0.3, 2.92)	1.88 (0.57, 5.94)	2.93 (0.97, 8.92)
9	23	0.35 (0.09, 1.36)	1.68 (0.75, 3.78)	3.64 (1.77, 7.31)	5.14 (2.6, 10.26)	0.2 (0.03, 1.08)	0.84 (0.19, 3.68)	1.59 (0.35, 7.02)	2.41 (0.6, 10.55)
10	17	1.18 (0.54, 2.61)	2.19 (1.02, 4.85)	5.04 (2.15, 11.62)	7.24 (2.89, 16.87)	0.65 (0.25, 1.71)	1.08 (0.38, 3.1)	2.2 (0.78, 6.45)	3.4 (1.25, 9.87)
10	20	0.77 (0.32, 1.8)	2.04 (1.18, 3.58)	4.3 (2.58, 7.21)	6.09 (3.7, 9.87)	0.43 (0.13, 1.38)	1.01 (0.33, 3.14)	1.9 (0.58, 6.04)	2.9 (0.96, 8.81)
10	23	0.49 (0.13, 1.87)	1.9 (0.84, 4.27)	3.71 (1.78, 7.45)	5.12 (2.62, 10.15)	0.28 (0.05, 1.51)	0.94 (0.22, 4.14)	1.61 (0.35, 7.19)	2.38 (0.59, 10.49)
11	17	1.39 (0.64, 3.03)	2.29 (1.07, 5.12)	5.07 (2.15, 11.75)	7.12 (2.86, 16.61)	0.78 (0.3, 2.09)	1.14 (0.4, 3.24)	2.22 (0.8, 6.45)	3.36 (1.22, 9.58)
11	20	0.99 (0.41, 2.32)	2.23 (1.27, 3.87)	4.34 (2.6, 7.31)	6 (3.63, 9.88)	0.55 (0.16, 1.81)	1.1 (0.36, 3.42)	1.9 (0.58, 6.14)	2.85 (0.94, 8.72)
11	23	0.71 (0.18, 2.69)	2.13 (0.94, 4.76)	3.75 (1.8, 7.58)	5.1 (2.58, 10.12)	0.4 (0.07, 2.18)	1.06 (0.24, 4.67)	1.63 (0.36, 7.26)	2.38 (0.58, 10.48)
12	17	1.67 (0.77, 3.66)	2.4 (1.1, 5.34)	5.11 (2.17, 11.67)	6.99 (2.75, 16.32)	0.93 (0.35, 2.47)	1.21 (0.41, 3.42)	2.2 (0.8, 6.47)	3.28 (1.2, 9.41)
12	20	1.28 (0.53, 3.07)	2.38 (1.39, 4.18)	4.38 (2.6, 7.32)	5.89 (3.6, 9.75)	0.72 (0.21, 2.36)	1.19 (0.38, 3.75)	1.9 (0.59, 6.11)	2.82 (0.93, 8.62)
12	23	0.99 (0.24, 3.92)	2.37 (1.04, 5.3)	3.79 (1.82, 7.69)	5.06 (2.55, 10.1)	0.56 (0.09, 3.09)	1.19 (0.27, 5.14)	1.65 (0.36, 7.42)	2.37 (0.58, 10.28)
13	17	2.01 (0.91, 4.34)	2.53 (1.17, 5.6)	5.12 (2.16, 11.69)	6.87 (2.72, 16.02)	1.12 (0.41, 2.92)	1.26 (0.44, 3.57)	2.21 (0.79, 6.52)	3.21 (1.17, 9.34)
13	20	1.67 (0.69, 3.99)	2.6 (1.5, 4.55)	4.41 (2.66, 7.37)	5.86 (3.58, 9.56)	0.93 (0.27, 3.08)	1.28 (0.42, 4.03)	1.93 (0.58, 6.12)	2.77 (0.92, 8.54)
13	23	1.4 (0.32, 5.73)	2.64 (1.16, 6)	3.85 (1.82, 7.85)	5.01 (2.57, 10)	0.78 (0.13, 4.32)	1.31 (0.29, 5.83)	1.67 (0.36, 7.34)	2.36 (0.57, 10.34)
14	17	2.37 (1.08, 5.19)	2.64 (1.24, 5.86)	5.15 (2.16, 11.72)	6.75 (2.67, 16.05)	1.33 (0.5, 3.51)	1.32 (0.46, 3.77)	2.23 (0.81, 6.54)	3.18 (1.15, 9.15)
14	20	2.17 (0.89, 5.26)	2.8 (1.63, 4.93)	4.44 (2.64, 7.4)	5.81 (3.52, 9.49)	1.2 (0.34, 4.04)	1.39 (0.45, 4.37)	1.94 (0.6, 6.21)	2.74 (0.91, 8.37)
14	23	1.95 (0.45, 8.1)	2.96 (1.3, 6.77)	3.9 (1.87, 7.89)	4.99 (2.51, 10.06)	1.1 (0.18, 6.37)	1.46 (0.33, 6.51)	1.69 (0.36, 7.45)	2.34 (0.57, 10.08)

[a] Years elapsed between high risk human papillomavirus detection and cervical cancer incidence assessment.

Web Table 8. Population–based surveys, International Agency for Research on Cancer.

**Country**	**Location**	**Survey year**
Bhutan	Thimphu, Lungthenphu	2011–2012 (1)
Nigeria	Ibadan	1999 (2)
Georgia	Tbilisi	2007 (3)
Guinea	Conakry	2006 (4)
Mongolia	Ulanbataar	2005 (5)
Nepal	Bharatpur	2006–2007 (6)
Pakistan	South Karachi	2006 (7)
Rwanda	Kigali	2013–2014 (8)
Vanuatu	Port Vila	2009–2010 (9)
Viet Nam	Ho Chi Minh City	1997 (10)

Web Figure 1. PANDORA Model - description of the method used to calculate the age-specific annual cervical cancer incidence rates (1993–2012) in high–risk human papillomavirus (HR HPV)–positive women in each location.

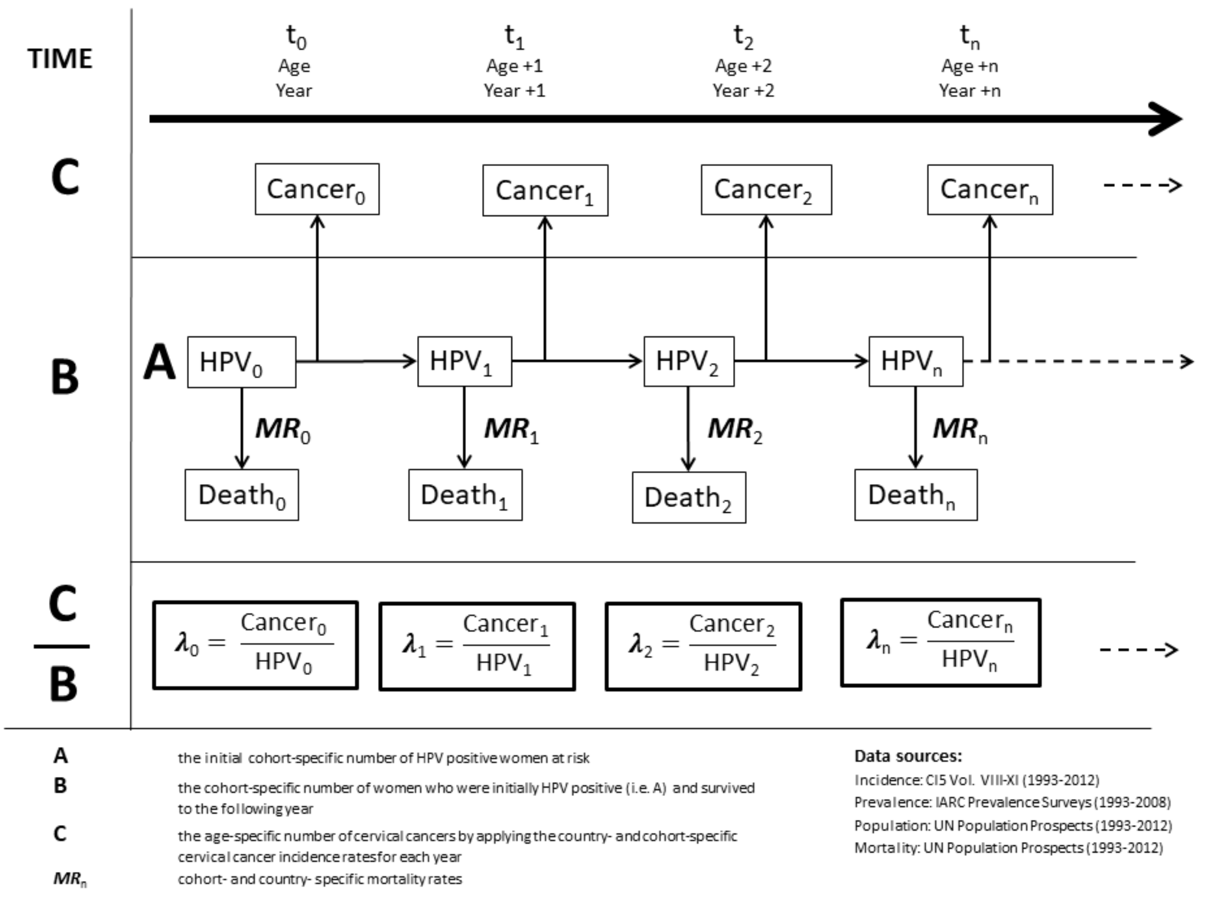

For each location, we calculated: A) the cohort-specific number of HR HPV-positive women for the year of HPV prevalence survey, by applying age-specific HR HPV prevalence measured in the survey to corresponding country-specific female birth-cohort, as reported in the UN world population prospects 2017 (11) B) the cohort-specific number of HR HPV-positive women for each following year since HPV measurement by applying the cohort- and country-specific mortality rates as reported in the UN world population prospects 2017 (12) and C) the cohort-specific number of cervical cancers, by applying the cohort-specific cervical cancer incidence rates obtained from CI5 to the female population of the corresponding birth-cohort and country as reported in the UN world population prospects 2017 (11). For each location and year since HPV measurement, we finally calculated the ratio between B and C to obtain the cohort-specific incidence of cervical cancer in HR HPV-positive women.

References

1. Tshomo U, Franceschi S, Dorji D, et al. Human papillomavirus infection in Bhutan at the moment of implementation of a national HPV vaccination programme. *BMC Infect Dis*. 2014;14:408.
2. Thomas JO, Herrero R, Omigbodun AA, et al. Prevalence of papillomavirus infection in women in Ibadan, Nigeria: a population-based study. *Br J Cancer*. 2004;90(3):638-645.
3. Alibegashvili T, Clifford GM, Vaccarella S, et al. Human papillomavirus infection in women with and without cervical cancer in Tbilisi, Georgia. *Cancer Epidemiol*. 2011;35(5):465-470.
4. Keita N, Clifford GM, Koulibaly M, et al. HPV infection in women with and without cervical cancer in Conakry, Guinea. *Br J Cancer*. 2009;101(1):202-208.
5. Dondog B, Clifford GM, Vaccarella S, et al. Human papillomavirus infection in Ulaanbaatar, Mongolia: a population-based study. *Cancer Epidemiol Biomarkers Prev*. 2008;17(7):1731-1738.
6. Sherpa AT, Clifford GM, Vaccarella S, et al. Human papillomavirus infection in women with and without cervical cancer in Nepal. *Cancer Causes Control*. 2010;21(3):323-330.
7. Raza SA, Franceschi S, Pallardy S, et al. Human papillomavirus infection in women with and without cervical cancer in Karachi, Pakistan. *Br J Cancer*. 2010;102(11):1657-1660.
8. Ngabo F, Franceschi S, Baussano I, et al. Human papillomavirus infection in Rwanda at the moment of implementation of a national HPV vaccination programme. *BMC Infect Dis*. 2016;16(1):225.
9. Aruhuri B, Tarivonda L, Tenet V, et al. Prevalence of Cervical Human Papillomavirus (HPV) Infection in Vanuatu. *Cancer Prev Res (Phila)*. 2012;5(5):746-753.
10. Anh PT, Hieu NT, Herrero R, et al. Human papillomavirus infection among women in South and North Vietnam. *Int J Cancer*. 2003;104(2):213-220.
11. United Nations, Department of Economic and Social Affairs, Population Division, World Population Prospects 2017 – online database: Population indicators. Accessed April 2, 2020, https://population.un.org/wpp/Download/Standard/Population/
12. United Nations, Department of Economic and Social Affairs, Population Division, World Population Prospects 2017 – online database: Mortality indicators. Accessed April 2, 2020, https://population.un.org/wpp/Download/Standard/Mortality/
